# Multiple mechanisms contribute to the development of clinically significant azole resistance in *Aspergillus fumigatus*

**DOI:** 10.3389/fmicb.2015.00070

**Published:** 2015-01-10

**Authors:** W. S. Moye-Rowley

**Affiliations:** Department of Molecular Physiology and Biophysics, Carver College of Medicine, University of IowaIowa City, IA, USA

**Keywords:** *Aspergillus fumigatus*, ABC transporter, azole resistance, cyp51A, abcG1

## Abstract

Infections caused by the filamentous fungus *Aspergillus fumigatus* are a significant clinical issue and represent the second most-common form of fungal infection. Azole drugs are effective against this pathogen but resistant isolates are being found more frequently. Infections associated with azole resistant *A. fumigatus* have a significantly increased mortality making understanding drug resistance in this organism a priority. The target of azole drugs is the lanosterol α-14 demethylase enzyme encoded by the *cyp51A* gene in *A. fumigatus*. Mutations in *cyp51A* have been described that give rise to azole resistance and been argued to be the primary, if not sole, contributor to azole resistance. Here, I discuss recent developments that indicate multiple mechanisms, including increased expression of ATP-binding cassette (ABC) transporter proteins, contribute to azole resistance. ABC transporters are well-established determinants of drug resistance in other fungal pathogens and seem likely to play a similar role in *A. fumigatus*.

## INTRODUCTION

Antibiotics have been one of medical science’s greatest advances but their continued efficacy is at risk. Over prescription and improper use has led to dramatic increases in resistant microorganisms with accompanying increased mortality. The problem of increased resistance is especially acute for fungal pathogens as the basic eukaryotic cell biology of these organisms limits the classes of antibiotics that avoid effects on the host.

*Aspergillus fumigatus* is the primary filamentous fungal pathogen of humans and an especially serious issue in any immunocompromised situation (Ruping et al., 2008). Azole drugs are most commonly deployed against *A. fumigatus* (and most fungi) as these drugs are well-tolerated and can be orally delivered. The success of azole antifungal therapy has naturally led to their extensive use both in the clinical setting and agriculturally, especially in Europe (discussed in Verweij et al., 2009). Strikingly, azole antifungals have been much more limited in their application in the United States (Pham et al., 2014) and this seems likely to help explain the differences between resistance mechanisms seen in these two regions. Increased azole resistance is a serious issue in treatment of aspergillosis. Mortality of azole-resistant aspergillosis can approach 90% (van der Linden et al., 2011).

Although the first azole resistant isolates of *A. fumigatus* were reported in the United States (Denning et al., 1997), understanding of the molecular basis of resistance was led by experiments from Europe. Early work on azole resistant isolates of *A. fumigatus* determined that these organisms contained mutations in one of the two genes (*cyp51A/B*) encoding the azole target enzyme: lanosterol 14α demethylase (Mellado et al., 2001). Focus on this gene (*cyp51A*) led to the surprising finding that the overwhelming majority of azole resistant strains contained two mutations and that both alterations were required for azole resistance (Verweij et al., 2007). This allele is referred to as TR34/L98H and consists of a 34 bp duplication in the promoter region linked to a replacement of leucine 98 with a histidine residue in the sequence of the enzyme (Mellado et al., 2007). This *cyp51A* allele confers multiazole resistance and has been found in resistant organisms ranging from Europe to India (Snelders et al., 2008; Chowdhary et al., 2012).

The origin of the TR34/L98H resistance allele as an environmentally selected variant is supported by several circumstantial lines of evidence. First, isolates from the soil led to the alarming discovery that 5–7% of these *A. fumigatus* strains contain this multiazole resistance lesion (reviewed in Chowdhary et al., 2013). Second, patients not previously exposed to azole drugs also exhibit aspergillosis with the TR34/L98H-containing fungus (Mellado et al., 2007). Third, genomic analyses of multiazole resistant *A. fumigatus* determined that strains containing the TR34/L98H allele were the most closely related compared to other azole-susceptible organisms, consistent with these drug resistant strains emerging from a single and recent alteration (Camps et al., 2012b). Finally, study of azole resistant organisms in the United States, where agricultural use of azoles is dramatically lower, has not uncovered the prevalence of the TR34/L98H *cyp51A* variant in drug resistant isolates (Pham et al., 2014).

While there is no question *cyp51A* mutations are important contributors to azole resistance, more recent studies have provided evidence that other resistance mechanisms are also at work in *A. fumigatus* (reviewed in Vermeulen et al., 2013). These other mechanisms have been observed in isolates derived from patients chronically exposed to azole drugs. This long term challenge with azole drugs drives generation of mutant spores that are now drug resistant. Since *A. fumigatus* exists in a multicellular state, acquisition of a mutant allele in one nucleus is very unlikely to produce a resistant organism. However, if a mutant spore is generated, then a resistant isolate will be produced. Analyses of these mutant strains of *A. fumigatus* that have been recovered after azole exposure have uncovered multiple new mechanisms of resistance to these antifungal drugs. Here I will review recent experiments that implicate the participation of ATP-binding cassette (ABC) transporter proteins and other mechanisms in azole resistance of clinical isolates.

## ABC TRANSPORTERS AND DRUG RESISTANCE IN PATHOGENIC FUNGI

The predominance of the TR34 L98H *cyp51A* allele in *A. fumigatus* represents an uncommon genetic distribution of azole resistance in other, better understood fungi. The pathogenic fungus for which we have the most detailed understanding of azole resistance mechanisms is the major human pathogen *Candida albicans*. Extensive analyses of the molecular basis for azole resistance in *C. albicans* led to the discovery of at least two different routes. The *C. albicans ERG11* gene encodes the lanosterol 14α demethylase in this fungus. Changes both in the sequence of the protein as well as alterations that increase *ERG11* transcription are associated with azole resistance (Perea et al., 2001; Morio et al., 2010). Increased expression of membrane transporter proteins is a second mechanism that synergizes with *ERG11* changes (reviewed in Morschhauser, 2010). Experiments in different clinical isolates (Selmecki et al., 2008; MacCallum et al., 2010) led to the finding that high level azole resistance required the cooperation of both the *ERG11* gene and a transcription factor encoded by the *TAC1* gene. The key role of Tac1 is to induce expression of ABC transporter proteins encoded by the *CDR1* gene along with other targets (Coste et al., 2004). These transporter proteins are of two different functional classes: the ABC transporters and major facilitator superfamily (MFS) transporters (reviewed in Prasad and Goffeau, 2012; Costa et al., 2014). Typically, these membrane proteins are found in the plasma membrane (PM) where they are thought to act as energy-dependent drug eﬄux pumps (Marger and Saier, 1993). Overproduction of the membrane transporters is usually due to amino acid substitutions in transcription factors that ultimately drive elevated mRNA level corresponding to these drug pumps.

This theme of elevated ABC transporter expression cooperating with Erg11 to confer azole resistance is seen in other pathogens including *Candida krusei* (Lamping et al., 2009) and *Candida glabrata* (Samaranayake et al., 2013). As is usual in instances of drug resistance, these multiple different mechanisms are engaged that work together to produce the full *in vivo* response to drug challenge. While the synergy of Erg11 and ABC transporters is well-described in *Candida* species, evidence implicating ABC transporters and other proteins in *A. fumigatus* has been less well-appreciated, likely owing in part to the elegant demonstrations tying *cyp51A* mutations to clinical azole resistance (Snelders et al., 2008). Recent experiments provide a rationale for reconsidering the importance of mechanisms beyond *cyp51A* in azole resistance in *A. fumigatus*.

## AZOLE RESISTANCE IN *A. fumigatus* MAY UTILIZE NON-*cyp51A*-DEPENDENT MECHANISMS

**FIGURE 1:**
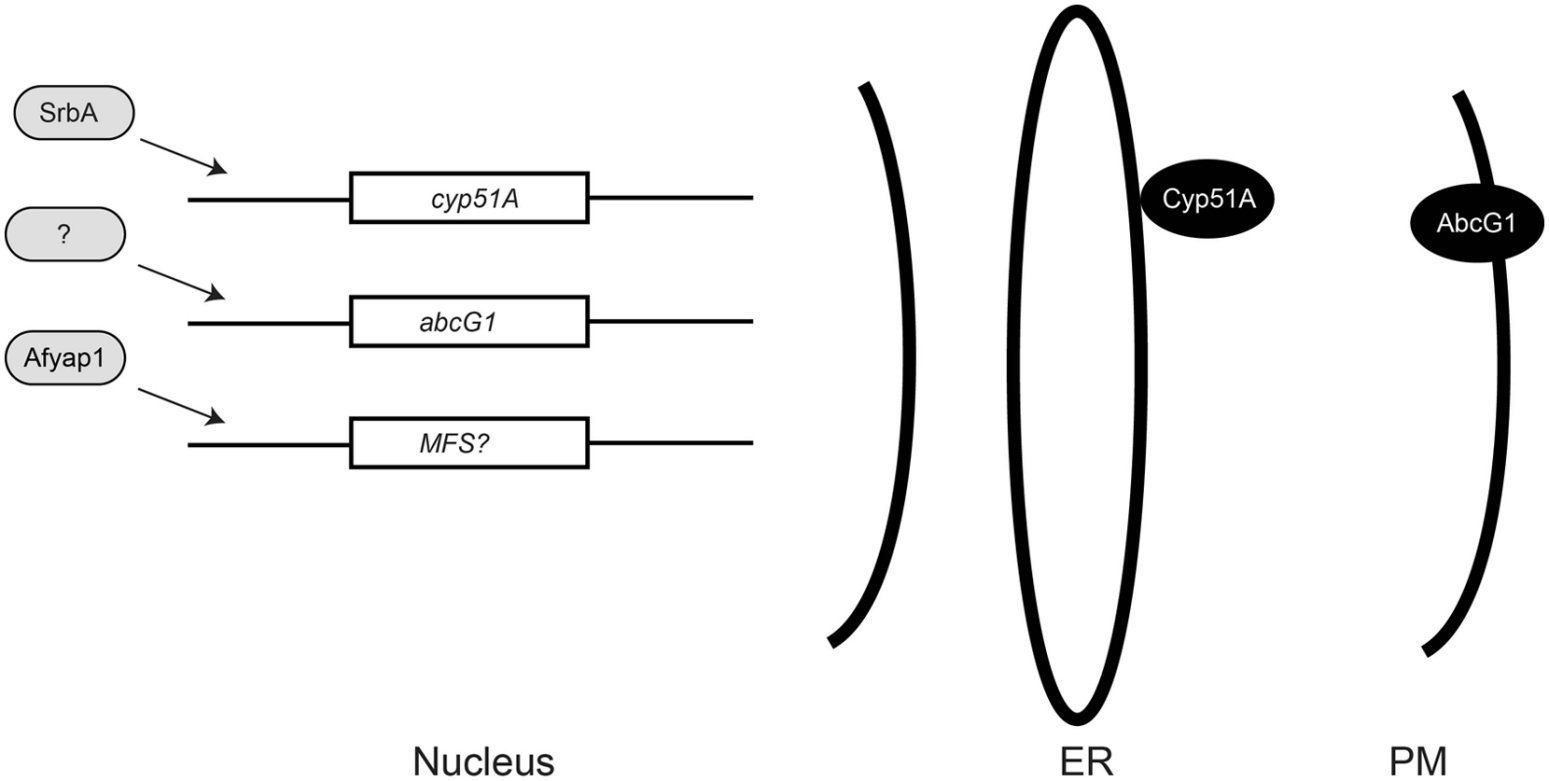
**Relationships of azole resistance determinants in *Aspergillus fumigatus*.** Known or putative transcription regulators are indicated in light gray as positive regulators of gene expression. The nuclear genes encoding the lanosterol α-14 demethylase (*cyp51A*), a plasma membrane (PM) ABC transporter (*abcG1*), or a major facilitator superfamily (MFS) transporter protein are indicated. Activation of an MFS-encoding gene by Afyap1 is still speculative at this point but is known to occur in other fungi (Alarco et al., 1997; Coleman et al., 1997; Rognon et al., 2006). The Cyp51A protein is docked to the endoplasmic reticulum (ER) by an N-terminal membrane spanning domain (Monk et al., 2014) while the AbcG1 protein is localized to the PM.

As discussed above, the large environmental reservoir of *cyp51A* azole resistant organisms has focused much attention on changes in this key target gene giving rise to drug resistant isolates (Verweij et al., 2009). Analyses of *A. fumigatus* azole resistant isolates from patient populations undergoing chronic azole exposure have provided important new information implicating other resistance pathways in antifungal resistance (**Figure 1**).

The first detailed study of azole resistant *A. fumigatus* strains that also contained wild-type *cyp51A* genes came from analyses of fungal isolates submitted to a regional mycology center at Manchester Hospital in the UK (Howard et al., 2009; Bueid et al., 2010). These investigators discovered that, between the years of 2004–2009, azole resistant isolates increased from 5 to 20%. While another center reported a similar increase in azole resistant *A. fumigatus* isolates (Mortensen et al., 2011), the unique feature of the Manchester data was the presence of a wild-type *cyp51A* gene in 43% of these resistant strains. These findings are consistent with more recent data from the United States in which a broad survey of *A. fumigatus* isolates found an overall lower frequency of azole resistance (5%) but 98% of these resistant isolates had no *cyp51A* lesion known to be associated with azole resistance (Pham et al., 2014). The origin of the *A. fumigatus* isolates in the UK and US studies were very different with the Manchester patients corresponding to patients with chronic aspergillosis and the US isolates coming from a wide range of sources (Pham et al., 2014). The primary commonality for the resistant isolates from these two different studies was the lack of *cyp51A*-dependent changes, strongly indicating the existence of other avenues of azole resistance.

The first data implicating ABC transporters in clinically relevant drug resistance has emerged quite recently. Before discussing these data, I would like to mention the confusing state of current nomenclature for ABC transporters in *A. fumigatus*. There is no consensus for gene names in this (or really any) family of protein in *Aspergillus* and I propose to adopt a system suggested earlier by Kovalchuk and Driessen (2010). This system employs a Human Genome Organization-approved scheme for naming ABC transporters based on their structural organization. I will use this nomenclature to refer to ABC transporters from *A. fumigatus* discussed here and encourage others to do the same.

A number of laboratories have demonstrated correlation between increased ABC transporter expression with associated azole resistance (Tobin et al., 1997; Slaven et al., 2002; Nascimento et al., 2003; da Silva Ferreira et al., 2006) but a functional link between the mRNA levels of a given ABC transporter and azole resistance remained elusive. Follow-up work on *A. fumigatus* isolates with a wild-type *cyp51A* gene led to the finding that mRNA encoding a particular ABC transporter was elevated in 8/11 azole resistant strains (Fraczek et al., 2013). This gene, referred to here as *abcG1* (aka *cdr1B* aka *abcB* aka AFUA_1G14330) encodes a homolog of the well-studied ABC transporters *Saccharomyces cerevisiae* Pdr5 and the *Candida* species Cdr1. Importantly, disruption of *abcG1* from one of the clinical isolates that overproduced this transcript led to a reduction in itraconazole MIC from >8 to 2 mg/L, demonstrating the functional requirement for this in itraconazole resistance (Fraczek et al., 2013).

Along with this work in clinical isolates, loss of *abcG1* was demonstrated to cause profound azole hypersensitivity in four different laboratory strains (Fraczek et al., 2013; Paul et al., 2013). Overproduction of the *abcG4* (aka *cdr1A* aka *abcA* aka AFUA_2G15130) gene product, that shares the highest degree of sequence conservation to abcG1, also elevated azole resistance. Fusions of green fluorescent protein to the C-terminus of either abcG1 or abcG4 indicated that the resulting fusion proteins were localized to the PM, consistent with their activity as ATP-dependent drug eﬄux pumps (Paul et al., 2013). While functional data implicating both of these ABC transporter proteins has been provided, these types of demonstrations remained to be accomplished for the large number (15) of *A. fumigatus* ABC transporters in the ABCG class of transporter proteins (Kovalchuk and Driessen, 2010).

While data are accumulating for the role of ABC transporters in azole resistance in *A. fumigatus*, other organisms also exhibit drug resistance that is triggered by changes in transcriptional regulators (recently reviewed in Paul and Moye-Rowley, 2014). As mentioned above, *C. albicans* Tac1 can acquire gain-of-function mutations that lead to enhanced activation of genes under its control (Coste et al., 2004). These target genes include the important azole resistance determinant *CDR1*. The work of Fraczek et al. (2013) suggests that similar mutations may be present in *A. fumigatus* azole resistant isolates overproducing *abcG1* but these remain to be found.

However, at least three examples of transcription factors that are important in azole resistance have been described. The first is the sterol response element binding protein (SREBP) from *A. fumigatus* referred to as SrbA (Willger et al., 2008). This regulator was discovered via its sequence conservation with SREBP from *Schizosaccharomyces pombe* and mammals (reviewed in Bien and Espenshade, 2010). Disruption of the *srbA* gene led to pronounced azole hypersensitivity, and perhaps most interesting, increased fluconazole susceptibility. This seems likely to be due to the decreased expression of *cyp51A* in the absence of SrbA (Willger et al., 2008) as *cyp51A*Δ strains exhibit a similar increased fluconazole susceptibility (Mellado et al., 2005). The importance of the trans-acting factor SrbA in expression of *cyp51A* suggests the possibility that mutants that increase SrbA activity might elevate azole resistance via their effect on a wild-type *cyp51A* locus. These type of mutant alleles of *srbA* have not been described at present.

A recently discovered azole resistance mechanism involving a transcription factor was described in an isolate from an immunodeficient patient undergoing chronic pulmonary aspergillosis (Camps et al., 2012a). Isolates recovered from early in the infection exhibited an azole susceptible phenotype that ultimately transitioned to azole resistant in time. Clever genetic analyses coupled with whole genome sequencing approaches detected a substitution mutation in the *hapE* gene as being responsible for the increased azole resistance. *A. fumigatus hapE* encodes a DNA-binding subunit of the Hap-complex that is a relative of the mammalian CCAAT-binding protein (see Dolfini et al., 2012 for a review). While a mechanistic explanation of the increased azole resistance driven by the mutant HapE-containing complex is still unknown, a potential contributor to this effect is provided by the finding that *cyp51A* expression is elevated in isolates bearing this lesion (Camps et al., 2012a).

A common link between resistance supported by changes in SrbA or HapE is the potential involvement of *cyp51A*. Azole resistant mutants with changes in these transcription factors could still possess a wild-type *cyp51A* allele yet exhibit elevated drug resistance. A final example that seems likely to be independent of *cyp51A* function is provided by the *A. fumigatus* homolog of *S. cerevisiae* Yap1. This protein is referred to as Afyap1 and is a basic region-leucine zipper transcription factor. ScYap1 is regulated by oxidative stress and normally resides in the cytoplasm due to rapid nuclear export (Kuge et al., 1997; Yan et al., 1998). Upon oxidative stress, nuclear export of ScYap1 is inhibited and the factor accumulates on target promoters, resulting in transcriptional induction of genes involved in redox regulation (reviewed in Morano et al., 2012). Afyap1 has been demonstrated to be regulated by oxidative stress at the level of nuclear localization and seems likely to be controlled by oxidants in a manner similar to that seen for ScYap1 (Lessing et al., 2007).

The relationship of Afyap1 to azole resistance was demonstrated by work using a truncation form of this protein (Qiao et al., 2010). The carboxy-terminal cysteine-rich domain (c-CRD) was deleted from Afyap1 and this mutant protein expressed from a multicopy plasmid in *A. fumigatus*. The c-CRD has been extensively studied in ScYap1 and serves as a negative regulatory site acting to exclude ScYap1 from the nucleus via interaction with the exportin protein Crm1 (reviewed in Guttler and Gorlich, 2011). The Afyap1 mutant lacking its c-CRD (referred to as TR Afyap1) was found to strongly elevate resistance to voriconazole but not influence itraconazole resistance (Qiao et al., 2010). A strain lacking Afyap1 was unaffected in terms of azole resistance but was highly sensitive to oxidants.

This behavior of Afyap1 is quite similar to that previously seen for both the *C. albicans* Yap1 homolog (Cap1) and ScYap1 (Alarco and Raymond, 1999). Loss of the Yap1-encoding genes from any of these organisms causes oxidative stress hypersensitivity but has no marked influence on azole sensitivity (Alarco et al., 1997; Alarco and Raymond, 1999). However, production of hypermorphic forms of Yap1 homologs does enhance azole resistance, typically through induction of expression of MFS-encoding gene expression (Alarco et al., 1997). Strikingly, in *S. cerevisiae*, while deletion of the gene encoding the major ABC transporter involved in azole resistance (Sc *PDR5*) led to a profound sensitivity to this drug (Sanglard et al., 1995), overproduction of ScYap1 fully suppressed this drug sensitivity (Alarco et al., 1997). This also seems likely to occur in *A. fumigatus* although further work will be required to confirm this possibility.

## SUMMARY

While early analyses of azole resistance in *A. fumigatus* were consistent with changes in the *cyp51A* gene being the primary if not sole driver of drug resistance, recent findings and a more nuanced view of resistance mechanisms do not support this simple picture. The key differentiator between the nearly exclusive involvement of *cyp51A* mutants in azole resistance and contribution of other pathways comes from consideration of the origin of resistant *A. fumigatus* isolates. An environmental reservoir of resistant organisms, certainly impacted if not completely driven by extensive use of azole-based agricultural fungicides, produced a large number of highly azole resistant isolates that were routinely discovered in patient populations (Verweij et al., 2009). During screening for azole resistant *A. fumigatus* appearing during chronic drug exposure, frequent isolates were found that contained wild-type version of *cyp51A* (Bueid et al., 2010). These resistant isolates indicate the presence of other resistance pathways in this filamentous fungus as seen in more extensively studied pathogens like the *Candida* species (Morschhauser, 2002).

The involvement of ABC transporters in *A. fumigatus* is most clearly indicated by the studies of Fraczek et al. (2013). Overproduction of *abcG1* was linked to multiple clinical isolates and shown to be required for azole resistance in one. The increasing number of azole tolerant isolates recovered from patients that exhibit no changes in the *cyp51A* locus supports the view that other mechanisms, such as ABC transporter overproduction, will be found to play important roles in azole resistance in *A. fumigatus*.

## Conflict of Interest Statement

The author declares that the research was conducted in the absence of any commercial or financial relationships that could be construed as a potential conflict of interest.
